# A review bridging pharmacokinetics, preclinical data, and human evidence on curcuminoids in cardiovascular disease

**DOI:** 10.3389/fphar.2026.1906759

**Published:** 2026-09-01

**Authors:** Katarzyna Zolnik, Andrzej Mogielnicki, Bartlomiej Kalaska

**Affiliations:** Department of Pharmacodynamics, Medical University of Bialystok, Bialystok, Poland

**Keywords:** bioavailability, blood pressure, cardiovascular disease, curcumin, curcuminoids, endothelial function, lipid profile, turmeric

## Abstract

Curcumin, the major curcuminoid of turmeric (*Curcuma longa* L.), has attracted sustained interest as a nutraceutical with pleiotropic anti-inflammatory, antioxidant, and vascular actions. However, extrapolating experimental *in vitro* findings to cardiovascular outcomes in humans is challenged by low aqueous solubility, chemical instability at intestinal pH, extensive first-pass metabolism, and substantial inter-product variability in formulation. Preclinical research supports antihypertensive and vasculoprotective effects, but many *in vitro* studies rely on micromolar exposures that are difficult to achieve following oral dosing. In humans, randomized trials and meta-analyses suggest modest improvements in surrogate cardiometabolic risk markers (e.g., minor alterations in lipid profiles and inflammatory biomarkers), with substantial heterogeneity across populations, doses, durations, and formulations. However, these results cannot establish turmeric-derived preparations as standard cardiovascular therapy. Evidence for clinically meaningful cardiovascular outcomes remains limited to a small number of short-term perioperative or acute coronary syndrome studies. For example, a single-center coronary artery bypass grafting trial reported fewer perioperative myocardial infarctions with high-dose curcuminoids, whereas in a larger perioperative trial nanocurcumin did not reduce postoperative atrial fibrillation. Overall, published GRADE assessments for cardiometabolic markers report low certainty. Exposure-guided, formulation-specific trials with standardized endpoints are required. In this mini review, we synthesize preclinical and human evidence on curcuminoids across key cardiovascular areas, including blood pressure and vascular function, lipid metabolism and inflammation, periprocedural myocardial injury and arrhythmias, and hemostasis. We also emphasize the importance of achieving systemic exposure, understanding metabolite biology, characterizing formulations, and using clinically relevant cardiovascular endpoints to improve the interpretability and reproducibility of future studies.

## 1 Introduction

Cardiovascular diseases remain a leading global cause of mortality and disability, with a rising absolute burden determined by population growth and aging (Roth et al., 2020). Despite major advances in prevention and pharmacotherapy, residual risk persists, and adjunct strategies targeting inflammation, oxidative stress, and metabolic dysfunction continue to be explored. Turmeric (*Curcuma longa* L.) contains a complex mixture of polyphenols and terpenoids, with curcuminoids (curcumin, demethoxycurcumin, bisdemethoxycurcumin) being considered its key bioactives (Yang et al., 2020; Wu et al., 2024). Curcumin has been studied across infectious and neoplastic disorders (Moghtaderi et al., 2017; Praditya et al., 2019; Obłoza et al., 2024), and its anti-inflammatory effects have motivated investigation in cardiometabolic disease. *In vitro* studies report modulation of angiotensin signaling, endothelial nitric oxide pathways, oxidative stress, and platelet activation. However, micromolar concentrations are frequently used in the experiments, potentially exceeding systemic exposures achievable after oral dosing (Heger et al., 2014; Nelson et al., 2017; Kroon et al., 2025).

Rather than adding another broad synthesis of curcumin’s cardiovascular or cardiometabolic effects, this mini review reframes the field through a translational pharmacology lens by asking how formulation-dependent systemic exposure, evidence from experimental cardiovascular models, and the hierarchy of human cardiovascular endpoints jointly shape the interpretability of the evidence. To achieve this, we integrate pharmacokinetic and formulation constraints with data from vascular, cardiac, and hemostatic models, as well as current human evidence. To support this narrative mini review, we performed a targeted, non-systematic literature search between November 2025 and May 2026 using PubMed/MEDLINE, PubMed Central, Crossref/DOI records, and publisher websites. Search terms combined curcumin/curcuminoids/turmeric-related free-text keywords, including “curcumin”, “curcuminoids”, “turmeric”, “*Curcuma longa*”, “nano-curcumin”, with cardiovascular, metabolic and hemostasis-related terms, including “cardiovascular disease”, “hypertension”, “type 2 diabetes”, “metabolic syndrome”, “dyslipidemia”, “blood pressure”, “endothelial function”, “lipid profile”, “platelet aggregation”, “antiplatelet”, “anti-thrombotic”, “coagulation”, “hemostasis”. We prioritized peer-reviewed English-language randomized controlled trials (RCTs), meta-analyses, evidence syntheses, and pharmacokinetic or formulation studies linking turmeric-derived preparations or curcuminoids to vascular, metabolic, cardiac, or hemostatic endpoints. Experimental studies were included when they clarified mechanisms, exposure–response relationships, or formulation-dependent translational issues relevant to cardiovascular interpretation. Studies were selected for narrative discussion based on relevance to the review’s translational focus, while Table 1 was restricted to RCTs with direct cardiovascular relevance and Table 2 to selected meta-analyses or evidence syntheses of cardiovascular risk markers. Most included studies were published between 2006 and 2025. A few older studies were included because they provided foundational data on curcumin pharmacokinetics, platelet aggregation, antithrombotic activity, and preclinical myocardial ischemia. Whenever available, we reported GRADE ratings from existing evidence syntheses (Guyatt et al., 2008).

**TABLE 1:** Key randomized human trials on curcuminoids in cardiovascular disease.

Setting	Study (year); N	Intervention and comparator	Follow-up duration	Primary outcome	Limitations
CABG	Wongcharoen et al. (2012); n = 121	Curcuminoids; 4 g/day; 3 days pre-op to 5 days post-op; placebo	33 days (primary outcome)	In-hospital MI: 13.1% vs. 30.0% (adj. HR 0.35, 95% CI 0.13–0.95; p = 0.038)	Single-center trial
CABG	Hossaini Alhashemi et al. (2022); n = 234	Nanocurcuminoids (SinaCurcumin); 240 mg/day; 3 days pre-op to 4 days post-op; placebo	At least 96 h	Postoperative AF: 11/113 (9.5%) vs. 14/121 (11.5%; p = 0.62)	Short perioperative exposure; no PK/exposure data
CABG	Tehrani et al. (2024); n = 80	Curcumin + piperine; 4-arm trial; 5 days post-op; placebo	5 days (majority of outcomes) 28 days (mortality)	Multiple primary outcomes. Mean change in placebo/500/1,000/1,500 mg/day: CRP 61.9/37.1/53.4/81.8 mg/L (p = 0.028); TAC 156.9/162.4/60.1/292.5 nmol/mL (p = 0.033); CK-MB 49.5/44.0/42.3/61.1 μg/L (p = 0.077); troponin I, LDH, EF, and AF not significant; 28-day mortality: 0/80	Biomarker-focused; multiple primary outcomes; underpowered for clinical outcomes
Unstable angina	Dastani et al. (2019); n = 40	Nanocurcuminoids (SinaCurcumin); 80 mg/day; 5 days; placebo	5 days	Primary: Progression to HF/STEMI; event numbers not reported. Secondary: Arrhythmia 9.5% vs. 29.4% (p = 0.207); EF change −0.58 vs. −0.88 (p = 0.738)	Short duration; not powered for clinical events
Coronary slow flow phenomenon	Rezaei et al. (2024); n = 50 randomized, n = 42 completed	Nanocurcuminoids (SinaCurcumin); 80 mg/day; 12 weeks; placebo	12 weeks	Primary/secondary outcomes not clearly specified; SAQ domains improved vs. placebo (p = 0.004–0.039); endocan, adropin, homocysteine, and lipid profile not significant	Small sample; patient-reported endpoint; no FMD or repeat angiography
PCI	Phrommintikul et al. (2019); n = 100	Curcuminoids; 4 g/day; at least 1 day before PCI to 1 day following PCI; placebo	At least 3 days	PCI-related myocardial injury: 32% vs. 38% (p = 0.675); peak hs-cTnT 201.0 ± 547.0 vs. 187.0 ± 703.9 ng/L (p = 0.912)	Short peri-PCI exposure; biomarker-defined endpoint
PCI	Aslanabadi et al. (2019); n = 110	Curcumin nano-micelles; 480 mg before PCI; standard care	1 day	Myocardial injury biomarkers at 24 h: CK-MB 21.8 vs. 23.1 U/L (p = 0.37), troponin I 0.18 vs. 0.22 ng/mL (p = 0.35)	Short duration; no blinding or placebo control

Abbreviations: AF, atrial fibrillation; CABG, coronary artery bypass grafting; CI, confidence interval; CK-MB, creatine kinase-MB; CRP, C-reactive protein; EF, ejection fraction; FMD, flow-mediated dilation; HF, heart failure; HR, hazard ratio; hs-cTnT, high-sensitivity cardiac troponin T; LDH, lactate dehydrogenase; MI, myocardial infarction; PCI, percutaneous coronary intervention; PK, pharmacokinetics; SAQ, Seattle Angina Questionnaire; STEMI, ST-segment elevation myocardial infarction; TAC, total antioxidant capacity.

**TABLE 2:** Selected meta-analyses and evidence syntheses relevant to cardiovascular risk markers.

Domain	Evidence synthesis	Summary of direction	Certainty assessment/limitations
Lipid profile	Dehzad et al. (2023)	Modest decrease in TC/TG/LDL-C; modest increase in HDL-C	GRADE: low; heterogeneity; formulation and population differences
BP	Hadi et al. (2019), Karimi et al. (2023)	Marginal SBP/DBP reductions in some analyses	Heterogeneous; depends on baseline BP and duration; overall low certainty
Vascular	Hallajzadeh et al. (2019)	Improvements in flow-mediated dilation; no effect on other endothelial markers	Small number of RCTs assessed
BP + vascular endpoints	Dehzad et al. (2024)	Overall, a small benefit on BP/endothelial-related endpoints	GRADE-assessed; overall limited certainty; subgroup effects possible
Umbrella overview	Xu et al. (2025)	Signals across multiple domains, including lipids and BP	Variable methodological quality; cardiovascular outcomes were not primary endpoints in most studies

Abbreviations: BP, blood pressure; DBP, diastolic blood pressure; HDL-C, high-density lipoprotein cholesterol; LDL-C, low-density lipoprotein cholesterol; RCTs, randomized controlled trials; SBP, systolic blood pressure; TC, total cholesterol; TG, triglycerides.

## 2 Chemistry, pharmacokinetics, and formulation

Curcumin is poorly soluble in water and undergoes rapid degradation and transformation depending on pH and matrix, which contributes to variable oral absorption (Kharat et al., 2017; Racz et al., 2022). After ingestion, curcumin is extensively metabolized (reduction and conjugation), and circulating unconjugated curcumin is typically low relative to conjugated metabolites. Moreover, reported concentrations may be affected by assay sensitivity and sample processing procedures (Heger et al., 2014; Kroon et al., 2025). Multiple strategies have been developed to enhance exposure, including co-administration with piperine (a uridine diphospho-glucuronosyltransferase inhibitor), phospholipid complexes (phytosomes), micelles, nanoparticles, and polymer-based delivery systems (Shoba et al., 1998; Khalil et al., 2013; Cicero et al., 2020; Stohs et al., 2020; Bateni et al., 2021; Khajeh Pour et al., 2023). These solutions complicate comparative interpretation because the same nominal dose can yield different systemic exposures depending on formulation, curcuminoid composition, and analytical verification (Nelson et al., 2017; Kroon et al., 2025). Therefore, it is important to distinguish between curcumin as a single compound, curcuminoid mixtures, and turmeric extracts, and to interpret claims in the context of systemic exposure (Anand et al., 2007; Hewlings and Kalman, 2017; Racz et al., 2022).

## 3 Preclinical evidence in cardiovascular models

Preclinical studies provide proof-of-concept for the vascular and cardiac actions of curcuminoids, although species differences and achievable exposure levels hinder clinical translation. Curcumin has been investigated for its effects on blood pressure (BP), vascular tone, myocardial stress, hemostasis, and thrombosis. In vascular smooth muscle cells (exposed to 0.01–10 µM curcumin) and an angiotensin II-induced hypertension model, curcumin downregulated angiotensin II type 1 receptor expression and attenuated agonist-dependent vasoconstriction (Yao et al., 2016). In nitric oxide-deficient hypertension (induced by N(ω)-nitro-L-arginine methyl ester), oral curcumin reduced systolic BP and partially restored endothelial responsiveness, with variable additional benefit from piperine co-administration (Hlavačková et al., 2011; Nakmareong et al., 2011). In an isoprenaline-induced myocardial ischemia model, combined pre- and post-treatment with curcumin improved electrocardiogram markers and myocardial ultrastructure, consistent with antioxidant and anti-inflammatory mechanisms (Manikandan et al., 2004). Curcumin also inhibited platelet aggregation *in vitro* (with half-maximal inhibitory concentrations ranging between 25 and 650 µM), with agonist-dependent potency and effects on eicosanoid metabolism, Ca^2+^ signaling, and platelet aggregation (Srivastava et al., 1995; Shah et al., 1999; Manikandan et al., 2004; Jantan et al., 2008; Chen et al., 2016). *In vivo* animal studies have reported antithrombotic or anticoagulant effects (Srivastava et al., 1985; Kim et al., 2012) and protection from endotoxemia-associated coagulopathy (Chen et al., 2007). These data highlight a possible antithrombotic effect, establishing a mechanistic basis for potential hemostatic interference.

While these preclinical models provide a theoretical basis for cardiovascular efficacy, their translational interpretation depends on whether the administered dose and route produce clinically significant systemic exposure. Most cited *in vivo* cardiovascular studies used oral or intragastric dosing, including 15 mg/kg in isoprenaline-induced myocardial ischemia, 50–100 mg/kg/day in N(ω)-nitro-L-arginine methyl ester-induced hypertension, and 300 mg/kg/day in Angiotensin II-induced hypertension. Using body-surface-area conversion, these doses approximate 0.17, 0.57–1.14, and 1.7 g/day, respectively, for a 70-kg adult (Reagan-Shaw et al., 2008), thereby nominally corresponding to human regimens summarized in Table 1, ranging from 80 to 240 mg/day nanocurcuminoids to 4 g/day conventional curcuminoids. However, dose overlap should not be interpreted as exposure equivalence. Systemic curcumin concentrations were generally not reported in these cardiovascular animal studies, whereas many proposed mechanisms derive from micromolar exposure (Yao et al., 2016), and direct antiplatelet activity required even higher micromolar concentrations in several assays. By contrast, conventional oral doses up to 8 g may result in undetectable serum curcumin, and recent formulation-comparison data indicate that unconjugated curcumin usually remains below 2 nM, with only transient low-nanomolar peaks (Lao et al., 2006; Kroon et al., 2025). Therefore, mechanisms requiring sustained micromolar parent curcumin, particularly direct platelet inhibition, are difficult to translate after oral supplementation unless metabolite activity, local deconjugation, tissue accumulation, or formulation-specific delivery is demonstrated. Consequently, extrapolating these mechanisms directly to human physiology requires significant caution.

## 4 Human evidence

### 4.1 Blood pressure and vascular function

Human trials evaluating turmeric-derived preparations and their influence on BP have produced mixed results, in part because many studies enrolled participants with near-normal baseline BP or concurrent antihypertensive therapy. In a 10-week, double-blind trial in patients with type 2 diabetes, curcuminoid supplementation did not significantly alter systolic or diastolic BP (Hodaei et al., 2019). Conversely, during a 14-week study, turmeric supplementation led to a decrease in both systolic and diastolic BP in diabetic patients (El-Rakabawy et al., 2025). While notable, these results must be interpreted cautiously due to the open-label design of the study. Enhanced formulations may show within-group improvements, but between-group differences are often small. In overweight subjects, a phytosomal curcumin preparation administered over 8 weeks decreased systolic BP in the intervention group, but end-of-study differences versus the placebo group were not significant (Cicero et al., 2020). In patients with metabolic syndrome, nanocurcumin did not meaningfully change BP despite improvements in some metabolic markers (Bateni et al., 2021). A pilot study in postmenopausal women suggested that combining endurance training with nanocurcumin intake leads to favorable changes in central arterial hemodynamics, supporting the potential vascular effects in this population (Sugawara et al., 2012).

Meta-analyses of RCTs generally report modest BP-lowering effects dependent on study duration and population, often characterized by heterogeneity (Hadi et al., 2019; Karimi et al., 2023). A meta-analysis regarding vascular endothelium demonstrated that curcumin significantly improves flow-mediated dilation, but it fails to alter other relevant surrogate biomarkers (Hallajzadeh et al., 2019). A recent GRADE-assessed dose-response meta-analysis further supported these findings, noting that the overall certainty of the evidence remains limited for BP and vascular surrogate endpoints (Dehzad et al., 2024).

### 4.2 Lipid metabolism, systemic inflammation, and cardiometabolic risk

To date, clinical evaluation of curcumin and other turmeric-derived preparations has focused primarily on intermediate cardiometabolic risk markers rather than significant cardiovascular endpoints. In patients with type 2 diabetes, curcuminoid supplementation reduced high-sensitivity C-reactive protein (hs-CRP) and increased adiponectin, with inconsistent effects on lipid fractions compared with the placebo group at study end (Adibian et al., 2019). Adiponectin is inversely associated with adipose tissue accumulation and is thought to exhibit anti-inflammatory and anti-atherosclerotic activity (Maeda et al., 2020). In an open-label trial, turmeric supplementation decreased malondialdehyde and tumor necrosis factor in diabetic patients (El-Rakabawy et al., 2025). In patients with metabolic syndrome, curcuminoids combined with piperine reduced hs-CRP and malondialdehyde, while increasing superoxide dismutase activity, supporting antioxidant and anti-inflammatory hypotheses (Panahi et al., 2015). A *post hoc* analysis of the same study population further revealed a significant reduction in circulating pro-inflammatory cytokines and chemokines (Panahi et al., 2016). However, such robust anti-inflammatory and antioxidant effects are not universally observed. In patients with unstable angina, a 5-day nanocurcuminoid administration failed to consistently alter myeloperoxidase, interleukin 18 or matrix metalloproteinase levels (Mohammad Pour et al., 2019).

Beyond mitigating systemic inflammation, addressing both impaired glucose metabolism and dyslipidemia is critical for reducing cardiovascular risk. In a 14-week trial, supplementation of turmeric capsules by diabetic patients did not alter fasting blood glucose or glycated hemoglobin levels (El-Rakabawy et al., 2025). In contrast, in a diabetes prevention study, curcuminoid extract reduced progression from prediabetes to type 2 diabetes (Chuengsamarn et al., 2012). A separate RCT reported that curcuminoid extract ameliorated atherogenic risk factors, including arterial stiffness, in patients with type 2 diabetes (Chuengsamarn et al., 2014). Moreover, nanocurcumin and phytosomal formulations have been associated with improvements in triglycerides and glycemic markers in some trials (Cicero et al., 2020; Bateni et al., 2021). In a large GRADE-assessed meta-analysis of 64 RCTs, turmeric/curcumin supplementation produced statistically significant but clinically modest reductions in total cholesterol, triglycerides, and low-density lipoprotein cholesterol, alongside increases in high-density lipoprotein cholesterol. However, the certainty of evidence across surrogate biomarkers was rated low (Dehzad et al., 2023). These findings suggest that curcumin may have a small beneficial effect on lipid profile in some populations, but it remains unclear whether this translates into a reduced risk of cardiovascular events.

### 4.3 Coronary artery disease

Although clinical evidence for curcumin or curcuminoids in coronary artery disease remains limited, these studies help assess its potential effects in acute conditions involving ischemia-reperfusion injury, inflammation, and oxidative stress. In coronary artery bypass grafting (CABG), a single-center double-blind RCT (n = 121) reported that curcuminoids at 4 g/day administered from 3 days preoperatively to 5 days postoperatively reduced the frequency of postoperative acute myocardial infarction (Wongcharoen et al., 2012). Although this change in cardiovascular event is clinically meaningful, the results should be confirmed in adequately designed and powered studies with rigorous evaluation of prespecified endpoints. Conversely, a larger perioperative trial utilizing a nanocurcuminoid formulation (SinaCurcumin; n = 234) failed to reduce the incidence of postoperative atrial fibrillation over 96 h of monitoring, nor did it significantly improve hs-CRP, malondialdehyde, or glutathione compared with placebo (Hossaini Alhashemi et al., 2022). A more recent CABG trial (n = 80) evaluating curcumin and piperine co-supplementation demonstrated favorable changes in selected inflammatory biomarkers, with no effect on clinically meaningful cardiovascular events, such as ejection fraction, mortality or atrial fibrillation (Tehrani et al., 2024). A short-term RCT in patients with unstable angina (n = 40) found no significant differences in arrhythmia incidence, progression to heart failure, or myocardial infarction with nanocurcuminoids compared with placebo (Dastani et al., 2019). These negative results highlight the limitation of small sample sizes and short follow-up for evaluating clinically meaningful outcomes. A 12-week RCT evaluating nanocurcuminoid intervention (SinaCurcumin; n = 50 randomized, n = 42 completed) in patients with coronary slow flow phenomenon failed to demonstrate significant changes in endothelial biomarkers, while at the same time reporting improvements in subjective, patient-reported angina symptoms (Rezaei et al., 2024). In the percutaneous coronary intervention with curcuminoids or nanocurcumin, pilot trials have failed to demonstrate a statistical reduction in biomarker-defined myocardial injury (Aslanabadi et al., 2019; Phrommintikul et al., 2019). While current human trials provide insight into specific, short-term perioperative settings, there is a notable absence of data regarding major cardiovascular outcomes. A comprehensive literature search did not identify RCTs evaluating the efficacy of curcuminoids or turmeric-derived preparations in the management of chronic heart failure, stroke, long-term secondary cardiovascular prevention or the reduction of major adverse cardiovascular events. Key randomized human trials with cardiovascular relevance are described in Table 1.

### 4.4 Hemostasis, platelet function, and thrombosis

Despite insights from *in vitro* and animal studies, human evidence investigating antithrombotic effects of curcumin or other turmeric-derived products remains limited. A randomized double-blind trial evaluating *Curcuma longa* L., *Angelica sinensis* (Oliv.) Diels, and *Panax ginseng* C.A.Mey. preparations reported minimal changes in standard coagulation parameters or platelet aggregation (Fung et al., 2017). The extent to which these findings apply to higher-dose curcuminoids or enhanced formulations is uncertain. Isolated case reports have documented bleeding events and/or international normalized ratio alterations associated with turmeric supplementation (Daveluy et al., 2014; Ghusn et al., 2023; Kalla, 2025). These, however, cannot be used independently to establish causality, as they can be prone to confounding. For patients with coronary artery disease, the clinically relevant issue is therefore not a proven independent bleeding risk of curcumin, but a potential interaction concern in the setting of antithrombotic pharmacotherapy. This distinction is important because such patients frequently receive aspirin, P2Y12 inhibitors, dual antiplatelet therapy, oral anticoagulants, or combined antithrombotic regimens. At present, the evidence hierarchy remains limited: mechanistic and experimental studies support possible modulation of platelet and coagulation pathways, isolated case reports provide only hypothesis-generating safety signals, and controlled comparative data demonstrating excess bleeding with curcuminoids added to antithrombotic therapy are lacking. This uncertainty is particularly relevant for high-bioavailability formulations or products combined with bioavailability enhancers, because systemic exposure may differ substantially between preparations (Shoba et al., 1998; Nelson et al., 2017; Kroon et al., 2025). Thus, any clinical recommendation should be precautionary rather than definitive. Given the widespread use of supplements among patients with coronary artery disease, future trials should prespecify hemostatic safety monitoring, bleeding definitions, antithrombotic co-medication reporting, and drug–supplement interaction assessment, particularly in patients receiving dual antiplatelet therapy, anticoagulants, or combined antithrombotic regimens.

## 5 Discussion

### 5.1 Safety, quality, and regulatory considerations

In RCTs, curcuminoids have generally been well tolerated, including short-term high-dose regimens (e.g., 4 g/day peri-CABG) (Wongcharoen et al., 2012). Nevertheless, supplement quality and content variability can undermine both safety and efficacy interpretation (Nelson et al., 2017; Racz et al., 2022). In contrast, evidence from observational studies presents a more complex safety profile. A clinicopathological case series has provided histological confirmation that turmeric supplements can induce liver injury (Papke et al., 2025). Moreover, registry-based causality assessment and the updated LiverTox monograph confirm that these supplements can rarely cause clinically apparent hepatotoxicity (National Institute of Diabetes and Digestive and Kidney Diseases, 2012; Halegoua-DeMarzio et al., 2023). Importantly, the risk may not be uniform across all products, and high-bioavailability formulations or piperine-containing products have been implicated in reported cases; however, comparative human data establishing a higher hepatotoxicity risk with these formulations remains limited (National Institute of Diabetes and Digestive and Kidney Diseases, 2012; Halegoua-DeMarzio et al., 2023). This supports the need for systematic adverse-event monitoring, liver function testing, and batch characterization in clinical studies. Beyond hepatotoxicity, the high prevalence of polypharmacy in patients with cardiovascular diseases requires careful assessment of potential drug–supplement interactions, especially when novel delivery systems or bioavailability-enhancing adjuvants, such as piperine, are used (Shoba et al., 1998; Nelson et al., 2017; Khajeh Pour et al., 2023).

### 5.2 Certainty of evidence

No cardiovascular outcome that has been formally assessed using GRADE currently reaches high certainty. This interpretation is consistent with a recent umbrella overview of intervention meta-analyses, which reported beneficial effects on selected surrogate outcomes, including lipid profile and BP, while emphasizing variable methodological quality, outcome heterogeneity, and limited direct evidence for clinically meaningful cardiovascular endpoints (Xu et al., 2025). It is essential to explicitly distinguish between changes in surrogate biomarkers and the prevention of clinically meaningful cardiovascular events. The most consistent clinical results are limited to surrogate cardiometabolic markers, while evidence for hard cardiovascular endpoints is sparse and heterogeneous. Importantly, the certainty ratings assigned to surrogate outcomes should not be extrapolated to clinical cardiovascular endpoints, which remain insufficiently studied. Meta-analyses and evidence syntheses relevant to cardiovascular risk markers are summarized in Table 2.

The relatively better-supported findings concern modest improvements in lipid profile parameters, including total cholesterol, triglycerides, low-density lipoprotein cholesterol, and high-density lipoprotein cholesterol, across multiple RCTs; however, in a meta-analysis that formally applied GRADE, the certainty of evidence for these surrogate outcomes was rated as low (Dehzad et al., 2023). Some evidence also suggests reductions in inflammatory and oxidative biomarkers, particularly hs-CRP and malondialdehyde, in selected populations, often when curcuminoids are administered together with piperine (Panahi et al., 2015; Adibian et al., 2019). To our knowledge, these outcomes have not been evaluated using GRADE, and therefore their certainty is interpreted narratively. Small reductions in BP have also been reported in some meta-analyses, although the findings are heterogeneous and supported by low GRADE certainty (Hadi et al., 2019; Karimi et al., 2023; Dehzad et al., 2024).

The less-supported areas mainly involve clinical cardiovascular endpoints and peri-procedural outcomes. A possible reduction in perioperative myocardial infarction after CABG has been reported in a single-center study (Wongcharoen et al., 2012). Evidence for a reduction in postoperative atrial fibrillation after CABG remains inconclusive, as a larger RCT of nanocurcumin did not demonstrate a significant effect on this endpoint (Hossaini Alhashemi et al., 2022). Similarly, evidence for effects on arrhythmias and heart failure outcomes in unstable angina is weak, as an underpowered short-duration trial failed to demonstrate benefit over the control group (Dastani et al., 2019). These clinical endpoints have not been systematically assessed using GRADE in the available literature, and conclusions regarding their certainty are therefore based on qualitative appraisal of study design, sample size, and consistency. Human evidence on coagulation and platelet function remains sparse and difficult to interpret, as available studies often involve mixed botanical formulations rather than isolated curcumin or curcuminoids (Fung et al., 2017).

### 5.3 Recommendations for future research

An exposure-guided trial design should recognize that many *in vitro* mechanisms require micromolar curcumin or curcuminoids exposure, whereas unconjugated curcumin’s/curcuminoid’s plasma concentrations after oral dosing usually remain in the nanomolar range (Heger et al., 2014; Kroon et al., 2025). Therefore, in order to establish the relationship between exposure and clinical response, trials should precisely specify and analytically verify the curcuminoid formulation, its curcumin or curcuminoid content, and, where feasible, circulating curcuminoids, metabolites, or conjugates (Nelson et al., 2017; Kroon et al., 2025). Because phytosomes, micelles, nanoparticles, and piperine co-administration can substantially modify systemic exposure and tissue distribution, studies should clearly differentiate between turmeric extracts, curcuminoid mixtures, and purified curcumin, and should report the manufacturing process, batch testing procedures, purity, batch-to-batch consistency, contamination risk, and stability conditions (Shoba et al., 1998; Cicero et al., 2020; Stohs et al., 2020; Khajeh Pour et al., 2023; Kroon et al., 2025).

Standardization and comparability also require CONSORT-compliant reporting, including prespecified primary and secondary endpoints and statistical analysis plans (Schulz et al., 2010). Endpoint selection should reflect study objectives, with prevention studies prioritizing validated vascular, inflammatory, and metabolic intermediate endpoints such as ambulatory BP, flow-mediated dilation, pulse wave velocity, lipid profile, and hs-CRP. Adequately powered trials should also consider pragmatic clinical outcomes, including hospitalization and major adverse cardiovascular events. In peri-procedural settings, standardized definitions of myocardial injury and clearly defined timing of biomarker measurements should be applied. Safety monitoring in cardiovascular populations should include systematic adverse-event reporting, liver function testing, and hemostatic parameters when clinically appropriate, and explicit assessment of potential interactions with antithrombotic therapy.

## 6 Conclusion

Curcuminoids remain biologically interesting nutraceuticals with documented preclinical pharmacodynamic effects relevant to vascular function, metabolism, and hemostasis. However, current clinical evidence supports only modest improvements in surrogate cardiometabolic markers, with findings limited by substantial heterogeneity and low certainty, as evaluated by published GRADE-assessed meta-analyses. Furthermore, formal certainty assessments are lacking, and the available evidence for clinically meaningful cardiovascular outcomes remains insufficient. Therefore, no recommendation for routine cardiovascular use can be made at this time. Future studies should be formulation-specific, adequately powered, exposure-guided, and based on clinically relevant endpoints to determine whether preclinical findings and improvements in surrogate biomarkers translate into cardiovascular outcomes.
